# Enhancement of parthenocarpy and fruit set through genome editing in tomato variety for processing use

**DOI:** 10.5511/plantbiotechnology.25.1003a

**Published:** 2026-03-25

**Authors:** Naohiro Koshi, Misaki Kobayashi, Hiroshi Ezura, Kenji Miura

**Affiliations:** 1 Graduate School of Life and Earth Sciences, University of Tsukuba, Tsukuba, Ibaraki 305-8572, Japan; 2 Tsukuba-Plant Innovation Research Center, University of Tsukuba, Tsukuba, Ibaraki 305-8572, Japan

**Keywords:** breeding, CRISPR-Cas system, genome editing, parthenocarpy, tomato for processing use

## Abstract

Tomatoes are extremely important plants that are cultivated worldwide, with various varieties grown in different regions. The traits required can vary depending on the region and intended use. Parthenocarpy, a trait that confers numerous advantages, reduces the labor required for pollination and minimizes the incidence of poor fruit set owing to temperature fluctuations. Mutations in *SlIAA9* induce parthenocarpy in tomatoes, and the introduction of this trait into processed varieties via genome editing suggests its potential to markedly shorten the breeding timeline. Genome editing has gained considerable attention as a breeding technique because it enables precise mutations in specific genes. However, only a few recent studies have reported examples of genome editing in Japanese tomato varieties for processing. In this study, we employed a genome-editing technique targeting *SlIAA9* to induce parthenocarpy in the Japanese tomato variety Natsunokoma for processing purposes, thereby reducing the labor required for pollination. The null-segregant *Sliaa9* mutant exhibited enhanced parthenocarpy and fruit set. These results suggest that improvements in fruit-bearing and parthenocarpic traits enhance the quality of tomato varieties that are mainly used for processing.

Tomatoes, which are native to South America, are the most widely cultivated vegetables worldwide. Although extensive breeding has improved traits such as color, size, flavor, yield, and disease resistance (Akotowanou et al. 2022; Scarano and Santino 2024), combining all desirable characteristics and adapting them to specific climates remains challenging.

Natsunokoma is a Japanese tomato variety that has been cultivated in Japan for >30 years as a valuable open-pollinated variety (Sato et al. 2004). Its features, such as determinate and jointless fruit stalks, are ideal for simultaneous harvesting, such as machine harvesting, and provide good flavor. It is crack-resistant and tolerant to Verticillium wilt, which facilitates its growth. Because it is an open-pollinated variety, it is reasonable to accumulate useful traits. The key traits of processing varieties include flavor, firmness, high yield, and reduced labor. However, fruit-setting can be affected by temperature, disease, and insect damage. Parthenocarpy, which allows fruit development without pollination (Ueta et al. 2017; Wang et al. 2005), is a crucial trait that results in high yields, controls cultivation conditions (Mubarok et al. 2023, 2024), and eliminates the need for labor (Knapp et al. 2017). For plants lacking parthenocarpy, artificial pollination methods such as insect-mediated pollination with bumblebees, vibration pollination, and hormone treatment are necessary for fruit formation during greenhouse cultivation (Hanna 2004). In processing tomatoes, labor-saving traits that reduce labor costs are very important. Several genes, including *pat-2* (Beraldi et al. 2004), *pat-k* (Takisawa et al. 2018), and *Sliaa9* (Wang et al. 2005), are associated with parthenocarpy; however, their application in tomato breeding remains limited.

*SlIAA9*, belonging to the Aux/IAA family, interacts with auxin response factors (ARFs) such as *SlARF7* to suppress fruit-set (Hu et al. 2023). When indole-3-acetic acid (IAA) levels are increased by pollination, SlTIR1 degrades SlIAA9 (Ben-Gera et al. 2012) to release ARFs and activate ARF-related genes. Deletion of the ARF-interacting domain of SlIAA9 results in the activation of a gene that is suppressed by *SlIAA9* and ARFs for the development of parthenocarpy (Hu et al. 2023).

The main method of plant breeding is hybrid breeding, but there are also methods such as mutation breeding and genetic modifications. “Natsunokoma” was also reported as a recipient cultivar to transfer the miraculin accumulation trait of transgenic “Micro-Tom” (Hiwasa-Tanase et al. 2022). Although hybrid breeding and mutation breeding are effective methods for improving traits and have become more efficient through genomic selection, they also have disadvantages, such as the time and cost required to obtain the desired traits by several backcrosses and the genetic background effects of the donor parent. Genetic modification can impart desired traits, but there remain concerns regarding its impact on ecosystems and social acceptability. In recent years, genome editing techniques have been developed for plant breeding (Scarano and Santino 2024). This method enables efficient and targeted trait introduction without altering other characteristics. In Japan, site directed nucleases (SDN)-1 genome editing has attracted attention because it is not subject to genetically modified organism (GMO) regulations (Tachikawa and Matsuo 2024). Even in tomatoes, which are considered model varieties with many examples of genome editing, there are fewer instances of the successful transfer of traits to commercial varieties than to model varieties. Specifically, Japanese varieties, other than those with high sugar content, remain largely understudied (Kawaguchi et al. 2021).

In this study, we used CRISPR/Cas9 technology to introduce the mutation in *SlIAA9* of Natsunokoma cells. The null-segregant *Sliaa9* mutant exhibited improved parthenocarpy and fruit set, highlighting its potential to enhance Japanese processing varieties with additional valuable traits.

*SlIAA9* (Solyc04g076850) contains seven exons and six introns. In the present study, we targeted the second exon using CRISPR/Cas9 (Supplementary Figure S1). In an initial experiment, when cultivated in a medium containing 100 mg l^−1^ kanamycin, the calli turned black, and necrosis occurred. It was considered that the Natsunokoma variety was highly sensitive to kanamycin. Therefore, a medium containing 50 mg l^−1^ kanamycin was used for the selection of pseudo-positive results. We selected 297 explants on kanamycin-containing callus-induction medium, from which 240 calli were obtained. Subsequently, 204 shoots were regenerated using the shoot induction medium. The regenerated shoots (Supplementary Figure S4) were subjected to PCR targeting the kanamycin resistance gene (*NPTII*) in the vector, and PCR-positive bands were detected in six individuals. These six cell lines were then transferred to rooting medium. PCR was repeated after two weeks, and the amplification products disappeared in five individuals (Supplementary Figure S2). In one individual in which the band remained, insertion of the genome editing cassette was confirmed by Southern blotting using the *NPTII* gene as a probe. This analysis indicated the presence of two cassettes (Supplementary Figure S3). At T0#82, when cassette integration was confirmed, a homozygous one-base-pair (bp) deletion was detected using Sanger DNA sequencing (Figure 1A). A deletion was observed 3–5 bp upstream of the protospacer adjacent motif (PAM) site. Although we could not determine the exact position owing to a continuous sequence of A residues, the location corresponded to a region frequently targeted by Cas9. This mutation introduced a premature stop codon 22 amino acids downstream, suggesting that the ARF binding motif was disrupted (Figure 1B). Both genome-editing cassettes were segregated in the T_1_ generation derived from self-pollination of T0#82. Additionally, because chromosome doubling typically occurs in tomato plants regenerated via tissue culture, the ploidy status of T0#82 was analyzed; consequently, this line was identified as diploid.

**Figure 1:**
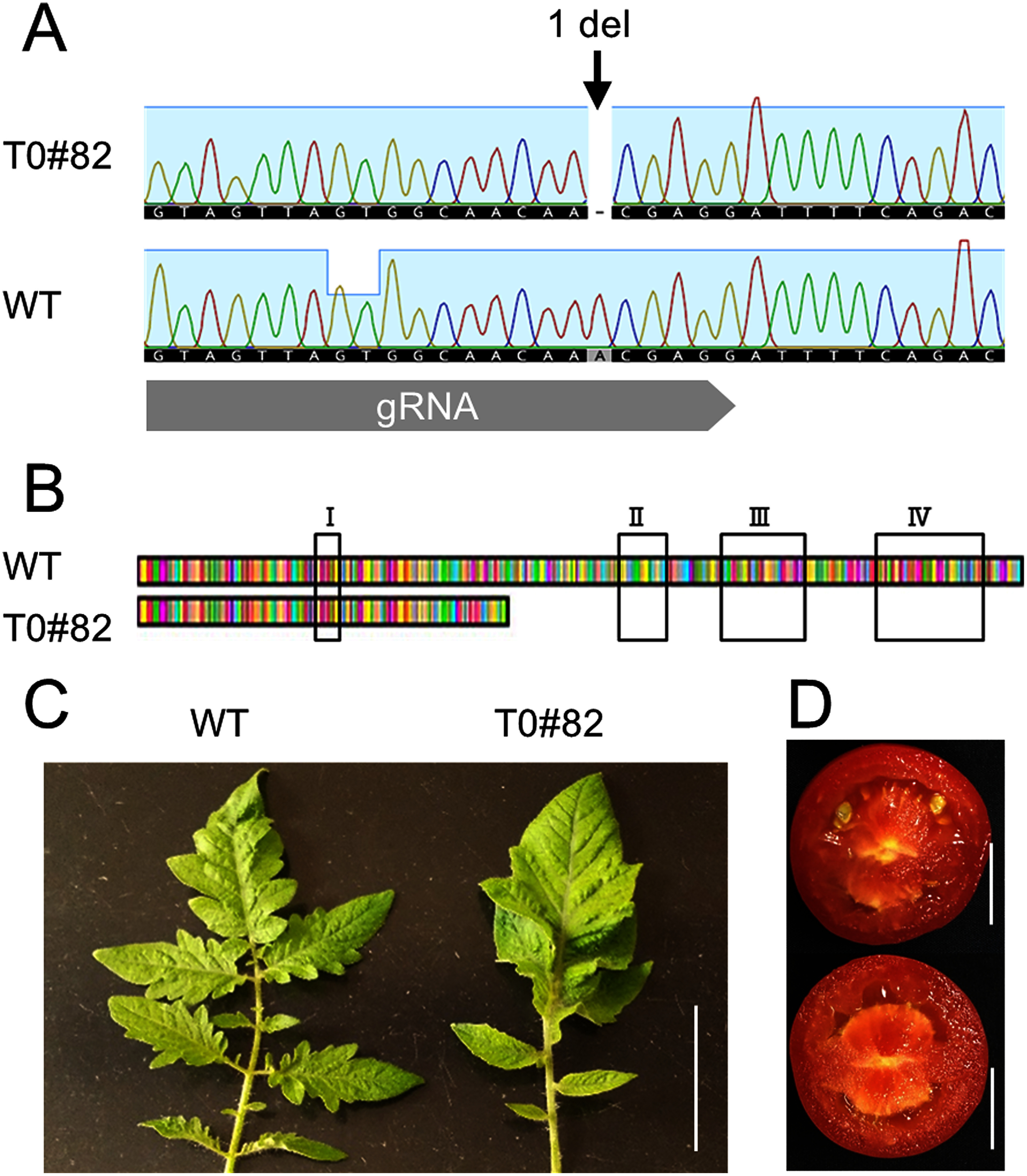
Figure 1. (A) Sanger DNA sequencing results of the target region in T0#82, showing a single-base deletion located 3–5 bases upstream of the PAM sequence. (B) A homozygous single-base deletion resulted in a premature stop codon 22 amino acids downstream of the mutation. Domain annotations: I, EAR motif (ethylene-responsive element binding factor-associated amphiphilic repression); II, SCF-mediated ubiquitin-proteasome degradation domain; III and IV, dimerization domains involved in interactions between Aux/IAA and ARFs. (C) Leaf morphology was altered in line T0#82. The previously serrated leaves became smoother due to the disappearance of serrations and the fusion of leaflets. The bar indicates 5 cm. (D) Cross-sections of fruits from T0#82. Emasculation was performed one day before anthesis. Pollination (top) or no pollination (bottom) was then performed. Bars indicate 1 cm.

The T_1_ seeds were obtained from T0#82 via artificial pollination. The one bp deletion detected in T0#82 was inherited by null-segregant T_1_ lines (Supplementary Figure S5). Altered leaf morphology was observed in T_1_ plants grown under controlled conditions. Serrated leaves were smoother in T_1_ plants because of the disappearance of serrations and the fusion of leaflets (Figure 1C). Parthenocarpy was also detected in the T_1_ plants. Emasculation was performed one day prior to anthesis, followed by pollination, and seeds were formed (Figure 1D). In contrast, fruits developed without seeds in the absence of pollination (Figure 1D). These results indicated that the *Sliaa9* mutation generated via genome editing altered leaf morphology and stimulated parthenocarpy. These phenotypes were consistent with those of a previous study (Osakabe et al. 2020). In the Natsunokoma cultivar, no significant differences in growth rate or plant height were observed between the T_1_ and T_2_ generations compared with the wild type (WT). Because this is a homozygous mutation, trait changes were stably inherited by subsequent generations.

The T_2_ generation was cultivated under controlled conditions to investigate self-fertilization. Even in the controlled culture room, approximately 40% of the *Sliaa9* mutant fruits (#82) exhibited parthenocarpy, whereas no parthenocarpy was observed in WT tomato fruits (Figure 2A, B).

**Figure 2:**
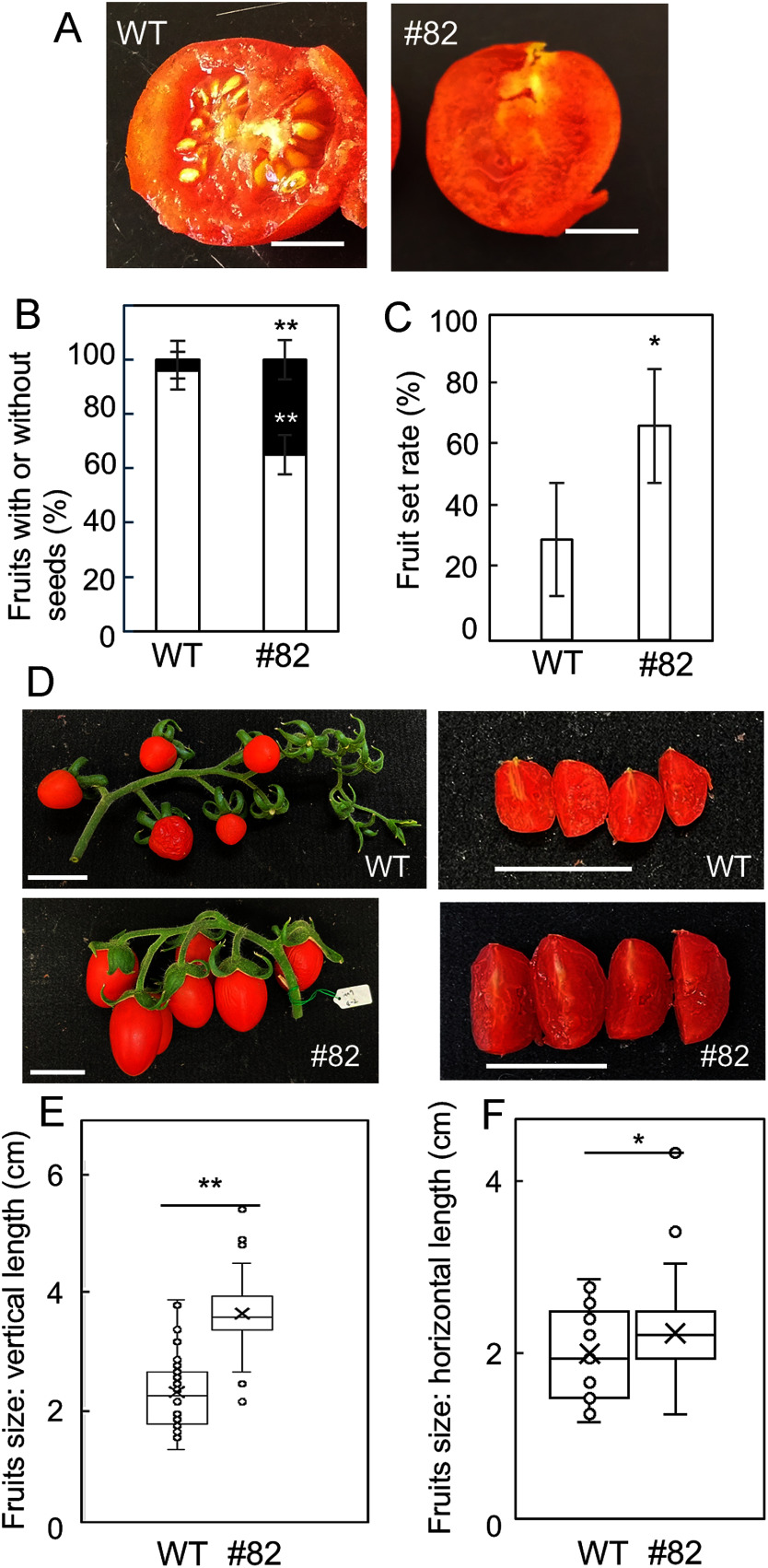
Figure 2. Fruit characteristics in *Sliaa9* tomato. (A, B) The numbers of seedless mature fruits developed without artificial pollination (parthenocarpic fruits, black bar) and fruits with seeds (white bar) from the WT and *Sliaa9* T_2_ line (#82) grown in a controlled cultivation room. Seven to 10 fruits were investigated to calculate the parthenocarpic percentage. Bars indicate 1 cm. Data are expressed as means±standard deviation (*n*=3). Statistical significance was determined using Student’s *t*-test of means for paired samples (** *p*<0.01). (C) Fruit-set from the first to the third clusters was determined and averaged. Significance was determined using Student’s *t*-test of means for paired samples (* *p*<0.05). (D) Photographs show representative clusters and cross sections of WT and line #82. Bars indicate 3 cm. (E, F) The fruit size of WT and line #82 was determined. Vertical and horizontal lengths were measured. Data are expressed as means±standard deviation (*n*≥28). Significance was determined using Student’s *t*-test of means for paired samples (* *p*<0.05, ** *p*<0.01).

Fruit setting and self-fertilization in the T_2_ generation were also compared in the greenhouse. During winter cultivation from January to April, the night temperature was set to approximately 5°C to increase pollination failure. Self-fertilization and fruit-set rates were evaluated without artificial pollination. Fruit set was improved in line #82 (Figure 2C), as the *Sliaa9* mutation enhanced parthenocarpy. Because low temperatures prevented fertilization, almost all tomato fruits of the WT and line #82 exhibited a parthenocarpic phenotype (Figure 2D). These results indicate that the genome-edited *Sliaa9* mutant enhanced parthenocarpy and fruit set. Furthermore, the fruit size of line #82 was larger than that of the WT (Figure 2E, F). The fruit size of the *Sliaa9* mutant did not decrease when parthenocarpic fruit production was promoted, leading to the suppression of yield reduction.

Genome editing has been widely studied as a novel method to improve crops in numerous plant species. (Atia et al. 2024; Mishra et al. 2024) In particular, its application in tomatoes has been extensively studied. (Larriba et al. 2024) For example, high-γ-aminobutyric acid tomatoes can be produced by deleting the autoinhibitory domain of SlGAD3, which is an important enzyme in the GABA biosynthesis pathway (Nonaka et al. 2017). This trait was introduced into the Sicilian Rouge variety and is commercially available from Sanatech Seed Co. (Tokyo, Japan). However, genome editing efficiency can vary among cultivars owing to differences in transformation and regeneration efficiencies (Nguyen et al. 2024; Stavridou et al. 2019). No previous genome editing attempts in Natsunokoma have been reported.

Unlike the Micro-Tom variety, Natsunokoma is highly sensitive to kanamycin or has a low efficiency of *Agrobacterium* infection as a varietal characteristic, making selection using kanamycin difficult. The callus induction and shoot regeneration rates were high (80.8% and 85%, respectively). The transformation efficiency remained low (0.49%). Alternative strategies, such as switching to hygromycin resistance (Sandhya et al. 2022) or using different *Agrobacterium* strains (Chetty et al. 2013), may improve efficiency.

A homozygous single bp deletion was confirmed in line 82, indicating that the CRISPR-Cas9 system also functioned in Natsunokoma. This mutation results in a premature stop codon in *SlIAA9*, resulting in the loss of domains III and IV, which are critical for ARF interactions. The enhancement of parthenocarpy significantly improved fruit set in a low-temperature environment, demonstrating that fruit production can be achieved without time-consuming and costly artificial pollination. This suggests the potential for a substantial reduction in labor during greenhouse cultivation. The yield of Natsunokoma was relatively high among processing tomato cultivars and exhibited disease tolerance (Satoh et al. 2004). The enhancement of fruit setting in these cultivars is expected to become more important in the future.

Natsunokoma possesses favorable traits, such as strong determinate and jointless fruit stems, making it suitable for mechanical harvesting and processing. The introduction of parthenocarpy demonstrates the potential of genome editing to efficiently improve traits. As most tomatoes used for processing in Japan are imported, domestically adapted, labor-saving varieties such as those that introduce parthenocarpy are in demand. Furthermore, in Japan, where cultivation areas are small, the incorporation of traits that enable more compact cultivation is desirable (Kwon et al. 2020). For the lines produced, it is also important to check yield potential and fruit quality, such as sugar and carotenoid content, when tomatoes for processing use are grown. Therefore, trait accumulation involving open-pollinated varieties is essential for future breeding strategies.

## Abbreviations

CRISPR: clustered regularly interspaced short palindromic repeats
IAA: indole acetic acid
PCR: polymerase chain reaction

## Description of supplementary files

Supplementary Figure S1. Design of gRNA target sites.

Supplementary Figure S2. PCR Analysis of kanamycin regeion for regenerated shoot.

Supplementary Figure S3. Verification of genome editing cassette copy number via Southern blotting.

Supplementary Figure S4. Process of plant regeneration.

Supplementary Figure S5. Comfirming null-segregants by PCR analysis for kanamycin resistance gene.

Supplementary Table S1. Sequence of primers for constructing vector and detection of variants.
