# Supplementary material for: Enhancement of parthenocarpy and fruit set through genome editing in tomato variety for processing use
Source: Plant Biotechnol (Tokyo). 2026 Mar 25;43(1):127–31. doi: 10.5511/plantbiotechnology.25.1003a (PMC13170819; doi:10.5511/plantbiotechnology.25.1003a)
Supplement: Supplementary Data [file plantbiotechnology-43-1-25.1003a-s001.pdf]

A

		CRISPR-P 2.0	CRISPR Direct (target site)		
Guide	Sequence	On-score	20 mer +PAM	12 mer +PAM	8 mer +PAM
guide001	GTAGTTAGTGGCAACAAACGAGG	0.8834	1	1	500

B

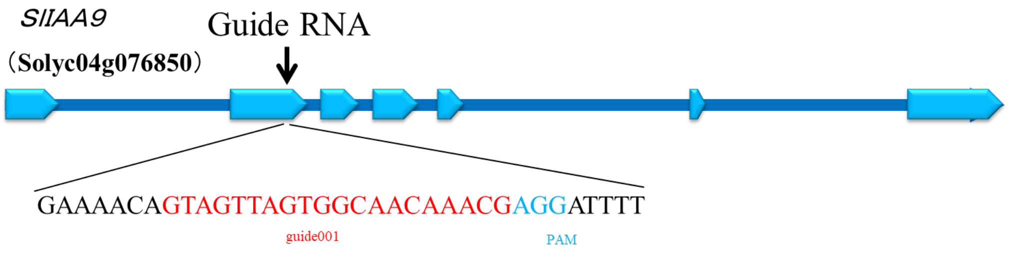

Supplementary Figure S1. Design of gRNA Target Sites. (A) A guide RNA was designed on the exon of SlIAA9 using the CRISPR-P 2.0 tool. Scores were calculated using both CRISPR-P 2.0 and CRISPR Direct web tools. (B) The structure of SlIAA9 mRNA and its exons is shown. The guide RNA was designed on exon 2.

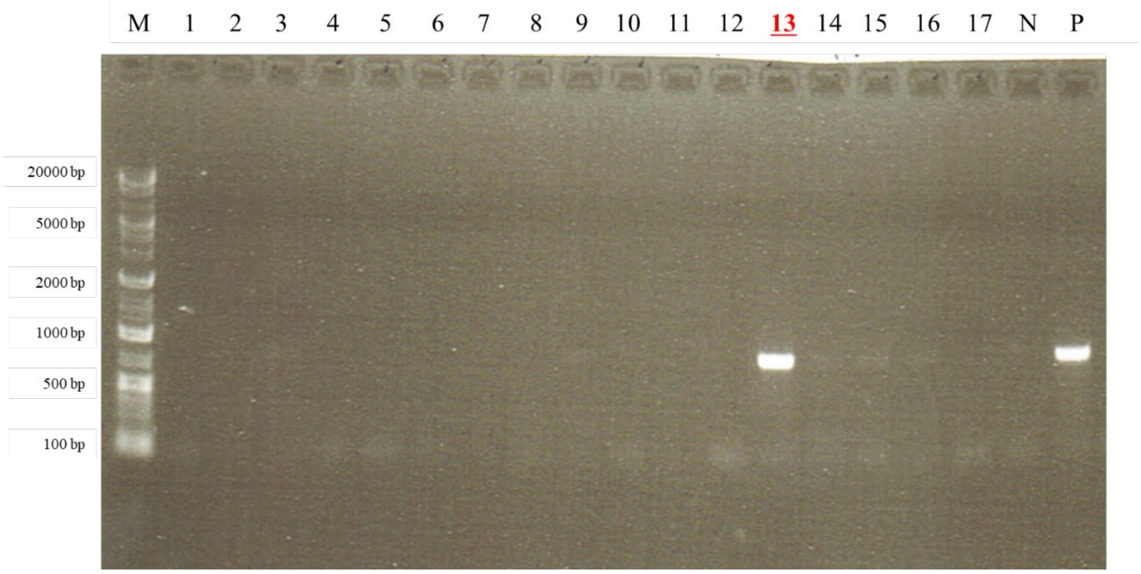

Supplementary Figure S2. PCR Analysis of kanamycin regeion for regenerated shoot. Amplification products were obtained using the primers NPTIICheck_Fw and NPTIICheck_Rev. T_0#82 was detected in lane13. Althogh thin band was detected in lane 3, 9, 14, 15, and 16, these band was false positive. M: Gene Ladder WideI, N: Negative Control, P: Positive Control.

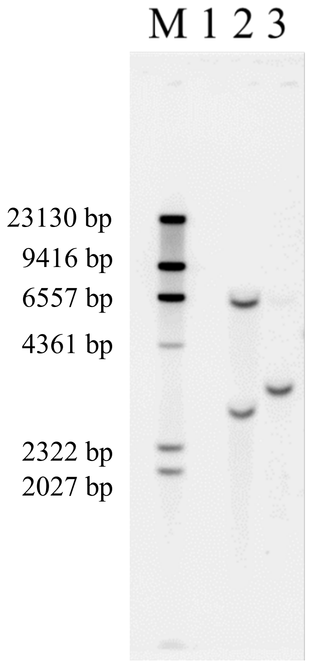

Supplementary Figure S3. Verification of genome editing cassette copy number via Southern blotting. The Southern blot analysis was performed with XbaI as the restriction enzyme and a probe targeting the kanamycin resistance region. A positive control, consisting of an individual confirmed to have a single cassette insertion, was included for comparison. M: Marker(DNA Molecular Weight Marker II, DIG-labeled), 1: WT (Negative Control), 2: T_0#82, 3: Positive Control

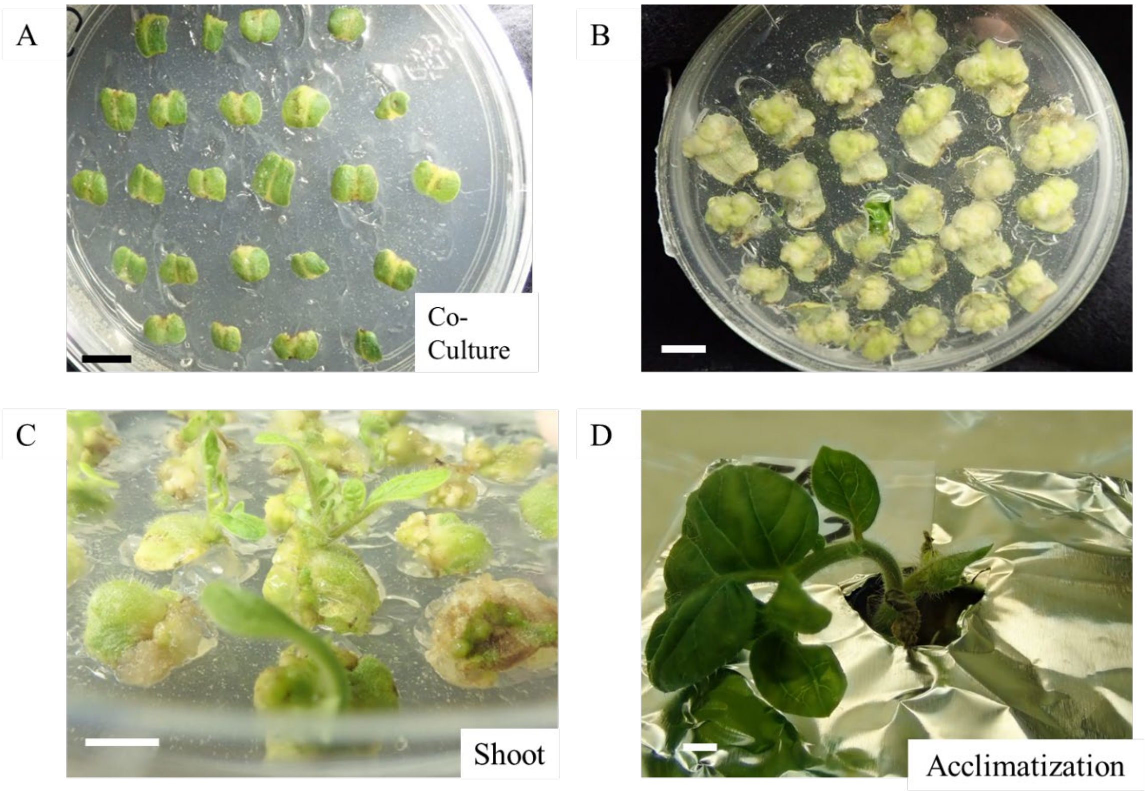

Supplementary Figure S4. Process of plant regeneration. (A) Explant placed in Co-Culture medium after inoculation with *Agrobacterium*. (B) Callus induction on C3 medium for 3 weeks. (C) Shoot induction on shoot medium for 1.5 months. (D) Acclimatization after root induction. Bars indicate 1 cm.

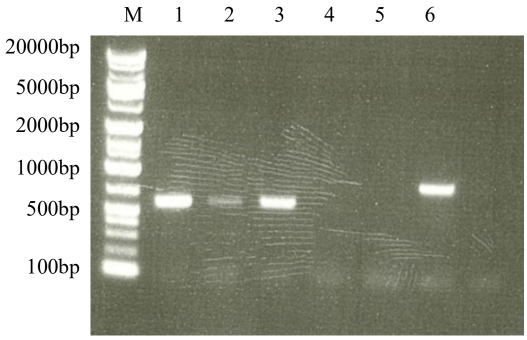

Supplementary Figure S5. Comfirming null-segregants through PCR analysis for the kanamycin resistance gene. Lane1-3: Sample quality was checked in the *Actin* region to assess PCR suitability using the primer sets Actin_Fw and Actin_Rev. Lane1: T_1 #13, Lane2: T_1 #13_2(from a different sampling part), Lane3: T_1 #1. Lane4-6: The *NPTII* gene (kanamycin resistance) was used to check for null-segregants with the primer sets NPTII Check_Fw and NPTII Check_Rev. Lane4: T_1 #13, Lane5: T_1 #13_2(from a different sampling part), Lane6: T_1 #1. Null individual was double checked by different sampling parts.

Supplementary Table S1. Sequence of primers for constructing vector and detection of variants.

Primer ID	Seq	Amplicon size	Template	Memo
guide001_Fw	ATTGGTAGTTAGTGGCAACAAACG		Solyc04g076850.1_guide001	guide RNA for IAA9 gene
guide001_Rev	AAACCGTTTGTTGCCACTAACTAC		Solyc04g076850.1_guide001	guide RNA for IAA9 gene
NPTIICheck_Fw	ATGATTGAACAAGATGGATTGCAC	792	pDe-Cas9-KAN	Induced check
NPTIICheck_Rev	TCAGAAGAACTCGTCAAGAAGGCG	792	pDe-Cas9-KAN	Induced check
IAA9_Fw	ATCTCAAGGCAACGGAGCTC		Solyc04g076850	Indel check by sequencing
IAA9_Rev	TTCGAGGCAGAGGCTAGAGT		Solyc04g076850	Indel check by sequencing
Actin_Fw	TAATAGAGTTGTAGGTAGTCTCGTG	474		
Actin_Rev	TAGTACTCTCATGATCCGAATCTTC	474		

Supplemental information

Materials and methods

Plant material

The tomato variety Natsunokoma (purchased from Tsurushinsyubyo) is a plum-type variety developed by the National Agriculture and Food Research Organization (NARO), with a fruit weight of approximately 50 g. It features a determinate type, non-layered fruit stalk, and is suitable for simultaneous harvesting.

Transformation

Transformation of genes to tomatoes was performed (Supplementary Figure S4; Sun et al. 2006). We incubated seeds and explants in a growth room at 25°C under 16 h light at 200 μmol m^{-2} s^{-1} and 8 h dark. 297 seeds were soaked in 70% ethanol for 2 min and treated with a bleach solution (40 ml distilled water and 5 ml of 10% commercial bleach) for 15 min in a rotator. The samples were then washed five times with sterile distilled water. The seeds were cultured for 8 d on 1/2 MS medium containing 3% sucrose. Shortly before the true leaves emerged, the cotyledons were divided into four parts, and the middle two parts were used as explants. The *Agrobacterium* solution was picked from the colonies, cultured in LB liquid medium supplemented with 100 mg l^{-1} ampicillin and 50 mg l^{-1} spectinomycin for 24 h, and centrifuged at 13,200 rpm for 30 s. After removal of supernatant, the bacterial pellet was resuspended in MS liquid medium supplemented with 1000×-diluted mercaptoethanol and 160 μM acetosyringone and adjusted to an optical density of 0.5.

The explants were incubated with *Agrobacterium* for 15 min immediately after preparation. Following infection, the cells were cultured in the dark for 5 d on a co-cultivation medium. The explants were then transferred to C3 medium (kanamycin 50 mg l^{-1}, augmentin 375 mg l^{-1}, zeatin 2 mg l^{-1}, 3 % (w/v) sucrose, and 0.8%(w/v) agar, pH 5.8) for callus induction, with the medium changed weekly. Next, the plants were further cultured in C2 medium supplemented with 50 mg l^{-1} kanamycin, 375 mg l^{-1} augmentin, 1.5 mg l^{-1} zeatin, 0.15 mg l^{-1} naphthaleneacetic acid, 3% (w/v) sucrose, and 0.8% (w/v) agar (pH 5.8) for 10 days (Park et al. 2003). Shoots were selected through culturing in SIM medium supplemented with 50 mg l^{-1} kanamycin, 375 mg l^{-1} augmentin, 1.0 mg l^{-1} zeatin, 0.05 mg l^{-1} indole acetic acid, 3% (w/v) sucrose, and 0.8% (w/v) agar at pH 5.8 for approximately 1.5 months. Shoots with visible growth points were cut and transferred to root induction medium containing 25 mg $^{-1}$l kanamycin, 375 mg l^{-1} augmentin, 1.0 mg

l^{-1} indole acetic acid, 3% (w/v) sucrose, and 0.8% (w/v) agar at pH 5.8. The rooted plants were then transferred to Rockwool and irrigated with liquid fertilizer (OAT-1 and OAT-2; Otsuka Chemical Co. Ltd., Osaka, Japan). The transgenic plants were irrigated using the same liquid fertilizer. Mutants were seeded through self-pollination, and the T_2 mutants were evaluated.

Vector construction and Agrobacterium

The pEn-Chimera and pDe-Cas9-Kan (Fauser et al. 2014) vectors for CRISPR/Cas9 were provided by Dr. Endo (NARO). We selected *SlIAA9* (Solyc04g076850.3) as a gene associated with parthenocarpy. After designing the target sequence using CRISPR-P 2.0, a sequence with a high on-score located within the domain structure and exon was selected. CRISPR Direct was used and guide001 was selected to minimize off-target effects (Figure 1).

The pEn-Chimera was digested with BbsI. The gRNA region (Supplementary Table S1), which was annealed by gradually lowering the temperature from 95°C to 40°C at a rate of 1°C min^{-1}, was inserted via ligation. The construct was transformed into *E. coli* DH5α. Successful insertion was confirmed via PCR. The plasmid was then incorporated into pDe-Cas9 using the Gateway system. The resulting vector construction was transformed into the *Agrobacterium* strain GV2260.

Detection of induced mutation

Leaf discs of approximately 5 × 5 mm were collected, frozen in liquid nitrogen, and ground using beads for shoots that had grown to approximately 1.5 cm after infection. DNA was extracted by adding 400 µl of DNA extraction buffer (1 M Tris-HCl, 5 M KCl, 0.5 M EDTA; pH 9.5), followed by centrifugation using a tabletop centrifuge. The supernatant was mixed with 300 µl of isopropanol using a vortex mixer. The supernatant was discarded after centrifugation at 13,200 rpm for 2 min. The resulting pellet was dried at 60°C for 5 min and subsequently dissolved in 100 µl TE buffer to prepare the sample solution.

PCR was performed in the kanamycin region of the vector using GoTaq® Green Master Mix (Promega). Positive shoots in which bands were detected (Supplementary Figure S2) were transferred to the rooting medium. For T_0#82, which developed roots, the sequence around the gRNA target site was confirmed using Sanger sequencing.

Plant growth conditions

The T_0 and T_1 generations were grown for 3 months in a climate-controlled room under a 25°C, 16 h light at approximately 200 µmol $m^{-2}s^{-1}$, and 8 h dark cycle, using liquid fertilizer (OAT-1 and OAT-2). Pollination was performed manually with fully mature pollen at the bud stage, 1-2 days before flowering.

The T_2 generation, in which the absence of the genome-editing cassette and the presence of the desired mutation were confirmed, was cultivated in a greenhouse from January to April. Plants were grown at ≥5°C, with six wildtype (WT) and six genome-edited mutants of the Natsunokoma cultivar.

Determination of fruit set and parthenocarpy

Fruit set was observed from the first to the second flower cluster without using artificial pollination or wind. The number of fruit stalks and fruits formed were recorded. To assess parthenocarpy, the fruits were harvested and dissected using a scalpel. The presence or absence of seeds was also assessed.

References

Fauser F, Schiml S, Puchta H (2014) Both CRISPR/Cas-based nucleases and nickases can be used efficiently for genome engineering in Arabidopsis thaliana. *Plant J* 79: 348–359

Park SH, Morris JL, Park JE, Hirschi KD, Smith RH (2003) Efficient and genotype-independent Agrobacterium-mediated tomato transformation. *J Plant Physiol* 160: 1253–1257

Sun HJ, Uchii S, Watanabe S, Ezura H (2006) A highly efficient transformation protocol for Micro-Tom, a model cultivar for tomato functional genomics. *Plant Cell Physiol* 47: 426-431

Southern EM (1975) Detection of specific sequences among DNA fragments separated by gel electrophoresis. *J Mol Biol* 98: 503–517
